# Different clinical phenotypes of a pair of siblings with familial hypercholesterolemia: a case report and literature review

**DOI:** 10.1186/s12872-023-03237-4

**Published:** 2023-05-01

**Authors:** Ze-Ping Wang, Ya-Jie Wu, Ying Gao, Jie Qian, Long-Tao Liu, Yuan-Lin Guo, Jian-Jun Li, Ke-Ji Chen

**Affiliations:** 1 Beijing University of Chinese Medicine, Beijing, 100029 China; 2 National Clinical Research Center for Chinese Medicine Cardiology, Xiyuan Hospital, China Academy of Chinese Medical Sciences, Beijing, 100091 China; 3 Cardiometabolic Medicine Center, Fuwai Hospital, Chinese Academy of Medical Sciences and Peking Union Medical College, Beijing, 100037 China

**Keywords:** Familial hypercholesterolemia, Low-density lipoprotein particle, Clinical phenotypic differences, Low-density lipoprotein cholesterol, Case report

## Abstract

**Background:**

Familial hypercholesterolemia (FH) leads to high plasma low-density lipoprotein cholesterol (LDL-C) levels and early cardiovascular morbidity and mortality. We treated a pair of siblings with FH. The cardiovascular manifestations in the proband were more severe than those in his elder sister, although they had almost similar LDL-C levels, ages, and lifestyles. Herein, we report the cases of this family to explore the possible causes of clinical phenotypic differences within the same genetic background.

**Case presentation:**

We treated a 27-year-old male patient and his 30-year-old sister, both with FH. The coronary angiogram in the male patient revealed 80, 70, and 100% stenosis of the initial, distal right coronary artery branch, and left anterior descending branch, respectively, whereas his sister had almost no coronary stenosis. We treated them accordingly and performed family screening. We found that the LDL-C/particle discordance of the proband is much greater than that of his elder sister. In addition, the average size of LDL-C particle in the proband was smaller than that in his sister.

**Conclusions:**

Patients with FH have a much higher risk of premature atherosclerotic cardiovascular disease, but the clinical manifestations are heterogeneous. The smaller LDL particle size may be the underlying cause for different clinical outcomes in this pair of FH cases and be a potential novel indicator for predicting the prognosis of FH.

## Background

Familial hypercholesterolemia (FH) is a common, inherited disorder of cholesterol metabolism that leads to high plasma low-density lipoprotein cholesterol (LDL-C) levels and early cardiovascular morbidity and mortality [1]. The prevalence of heterozygous familial hypercholesterolemia (HeFH) has been estimated to be 1/500–1/200 in most ethnic groups worldwide [2, 3]. Early and adequate treatment can eliminate or decrease the lifetime risk of coronary heart disease (CHD) associated with high levels of LDL-C due to FH. Although, statins have been confirmed to effectively reduce LDL-C levels, most patients with FH require more than a single medication to sufficiently lower LDL-C. The addition of ezetimibe and proprotein convertase subtilisin/kexin type 9 (PCSK9) inhibitor, a new medication for lowering LDL-C by decreasing the degradation of LDL receptors (LDLRs), is recommended by the newest European Society of Cardiology/European Atherosclerosis Society guidelines [4, 5]. For those with poor response to lipid-lowering drugs, LDL apheresis is an important treatment modality to remove LDL from circulation [6, 7].

Patients with compound HeFH or homozygous FH have extremely high and difficult-to-control LDL-C levels, which can lead to severe atherosclerotic cardiovascular disease (ASCVD) events even in their youth, including during adolescence [3]. Therefore, when a proband is clinically diagnosed with FH clinically, regardless of genetic evidence, a screening and early treatment for family members should be started as soon as possible. We treated a male patient with FH and three of his affected relatives and drew a family tree. Interestingly, cardiovascular manifestations in the proband were quite severe than those in his elder sister, despite having comparable LDL-C levels, age, and lifestyles. The coronary angiogram in the male patient revealed 80, 70, and 100% stenosis of the initial right coronary artery (RCA) branch, distal RCA branch, and left anterior descending (LAD) branch, respectively, whereas his sister had almost no coronary stenosis. Therefore, this study aimed to explore the possible causes of the observed clinical phenotypic differences within the same genetic background in this family.

## Case presentation

**Fig. 1:**
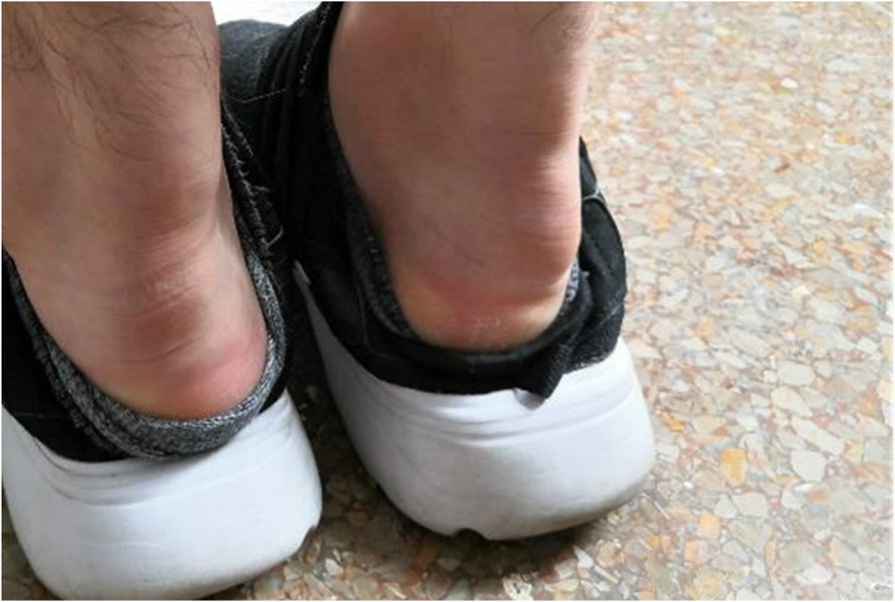
Significant Achilles tendon xanthoma of the proband

A 27-year-old male patient with typical symptoms of angina pectoris was admitted to the lipid ward of Fuwai Hospital in June 2020. He was 180 cm tall and weighed 72 kg; he had no history of diabetes and hypertension. He had been experiencing swelling and pain in his lower limb joints since 2010; furthermore, laboratory examinations revealed that his plasma total cholesterol (TC) was as high as 11–12 mmol/L. He did not take any lipid-lowering medications except simvastatin for one week, and the pain in his lower limb joints had persisted for the past 10 years. Typical symptoms of angina pectoris appeared in 2019 and became worse gradually since then. His fasting plasma LDL-C level determined at Fuwai hospital was 10.68 mmol/L without taking any lipid-lowering medication intake. Furthermore, notable xanthoma of the Achilles tendon (Fig. 1) and corneal arch could be observed. Ultrasound revealed multiple carotid plaques and thickening of the right carotid intima-media. Coronary computed tomographic angiography (CCTA) showed atherosclerotic lesions in the aorta, multiple occlusions in the distal LAD, and significant stenosis in the left circumflex artery and RCA.

**Fig. 2:**
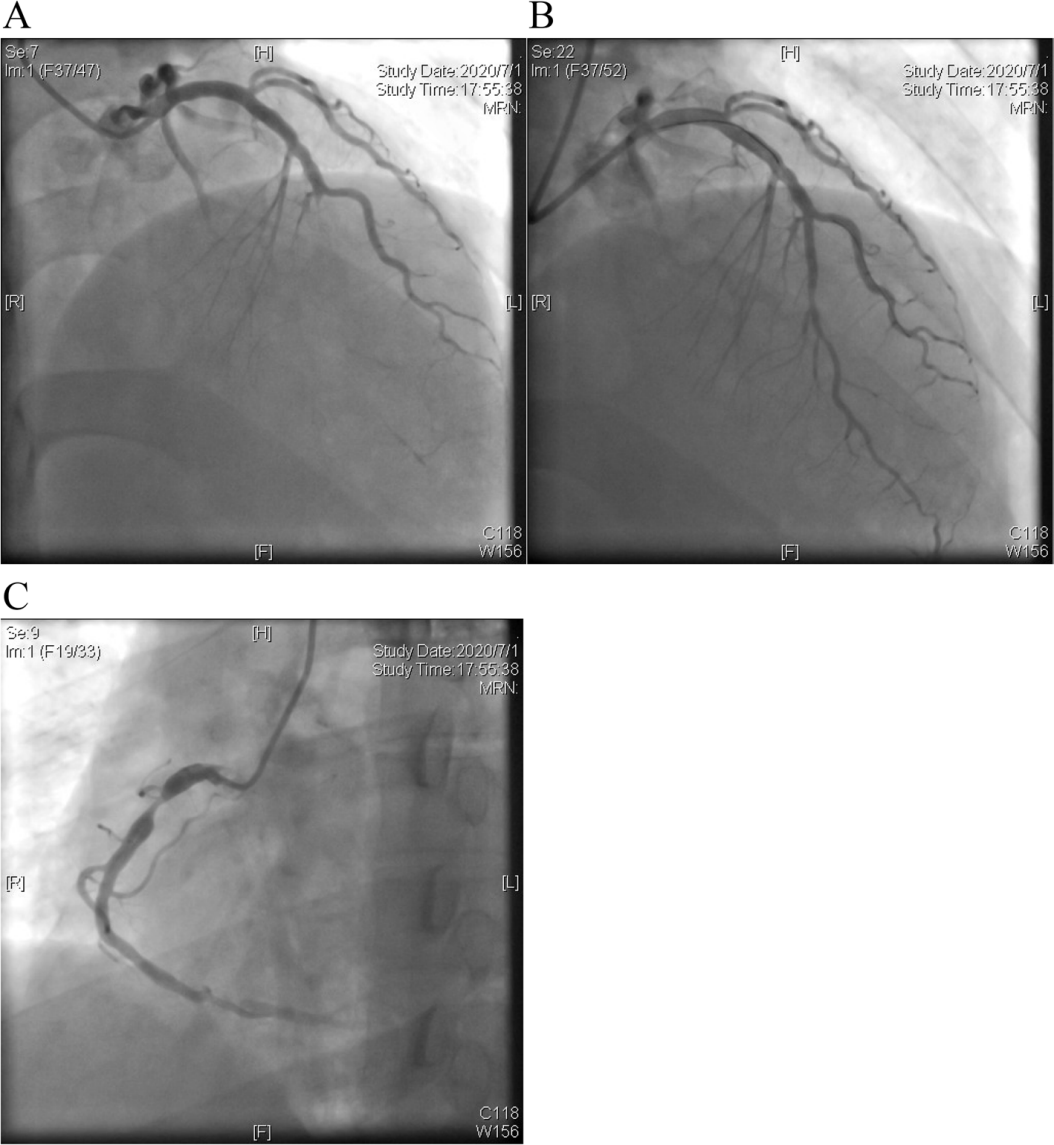
Percutaneous coronary intervention on July 1, 2020. **A** Totally occlusion of LAD. **B** After stent implantation in LAD. **C** Significant stenosis of RCA. LAD, left anterior descending artery; RCA, right coronary artery

He was diagnosed with FH based on the Dutch Lipid Clinic Network (DLCN) with a score of 17 (eight points for LDL-C > 8.5 mmol/L; six points for xanthoma of the Achilles tendon; two points for premature CHD; and one point for hypercholesterolemia family history). Rosuvastatin (20 mg/day) and ezetimibe (10 mg/day) were administered as the initial therapy. CCTA showed a coronary calcium score (CAC) of 125 and severe stenosis of the LAD and RCA. Further percutaneous coronary angiography revealed 100% occlusion in the LAD (Fig. 2A), and 80% stenosis in the proximal and 70% stenosis in the distal segment of RCA (Fig. 2C). A Promus Premier stent (2.25 mm × 16 mm) was placed at the LAD (Fig. 2B) on July 1, 2020 after a rapid LDL-C reduction by lipoprotein apheresis on June 29, 2020.

To further lower the plasma LDL-C levels, intensive treatment consisting of subcutaneously administered evolocumab 420 mg every month and oral pharmacotherapy was recommended after discharge. However, the patient reduced the dose of evolocumab to 140 mg every 2 weeks due to personal economic reasons in the next 3 months (Table 1).

**Table 1:** Blood lipid levels and lipid-lowering strategies of the proband

Date	TC	LDL-C	Lp(a)	Lipid-lowering strategies	Notes
6-24-2020	12.79	10.68		rosuvastatin 20 mg qd ezetimibe 10 mg qd	baseline levels without any medication
6-29-2020	10.07 (−21.3%)	8.31 (−22.2%)	49.1	rosuvastatin 20 mg qd ezetimibe 10 mg qd	just before LA
6-29-2020	3.29 (−74.2%)	2.56 (−76.0%)	14.2 (−71.1%)	rosuvastatin 20 mg qd ezetimibe 10 mg qd LA	immediately after LA
6-30-2020	3.81 (−70.2%)	2.78 (−74.0%)	20.1 (−59.1%)	rosuvastatin 20 mg qd ezetimibe 10 mg qd	
7-1-2020				rosuvastatin 20 mg qd ezetimibe 10 mg qd	PCI at LAD
7-3-2020	4.59 (−64.1%)	3.35 (−68.6%)		evolocumab 420 mg SC rosuvastatin 20 mg qd ezetimibe 10 mg qd	
7-7-2020	3.83 (−70.1%)	2.79 (−73.9%)		rosuvastatin 20 mg qd ezetimibe 10 mg qd	
7-14-2020	3.57 (−72.1%)	2.22 (−79.2%)	30.6 (−37.7%)	rosuvastatin 20 mg qd ezetimibe 10 mg qd	15 days after LA and 11 days after evolocumab 420 mg
7-27-2020	4.5 (−64.8%)	2.93 (−72.6%)	18.1 (−63.1%)	rosuvastatin 20 mg qd ezetimibe 10 mg qd	
8-17-2020	5.21 (−59.3%)	3.49 (−67.3%)	39.5 (−19.6%)	rosuvastatin 20 mg qd ezetimibe 10 mg qd	
9-14-2020	5.36 (−58.1%)	3.66 (−65.7%)	40.9 (−16.7%)	rosuvastatin 20 mg qd ezetimibe 10 mg qd	
9-19-2020				evolocumab 140 mg SC rosuvastatin 20 mg qd ezetimibe 10 mg qd	
10-13-2020				evolocumab 140 mg SC rosuvastatin 20 mg qd ezetimibe 10 mg qd	
10-17-2020				evolocumab 140 mg SC rosuvastatin 20 mg qd ezetimibe 10 mg qd	
10-20-2020	4.28 (−66.5%)	2.81 (−73.7%)	25.5 (−48%)	rosuvastatin 20 mg qd ezetimibe 10 mg qd	
3-9-2021	7.18 (−43.9%)	5.67 (−46.9%)	24.7 (−49.7%)	rosuvastatin 20 mg qd ezetimibe 10 mg qd	
9-27-2021	6.15 (−51.9%)	4.70 (−56.0)		rosuvastatin 20 mg qd ezetimibe 10 mg qd	
10-26-2021	4.63 (−64.6%)	3.17 (−70.3%)	22.6 (−54.0%)	rosuvastatin 20 mg qd ezetimibe 10 mg qd	
10-27-2021				rosuvastatin 20 mg qd ezetimibe 10 mg qd	PCI at RCA

The levels and percent of change from baseline are expressed as mmol/L (%) for TC and LDL-C, and mg/dL (%) for Lp(a)

*TC* Total cholesterol, *LDL-C* Low density lipoprotein cholesterol, *Lp(a)* Lipoprotein (a), *LA* Lipoprotein apheresis, *PCI* Percutaneous coronary intervention, *qd* Once a day, *RCA* Right coronary artery, *SC* Subcutaneous

**Fig. 3:**
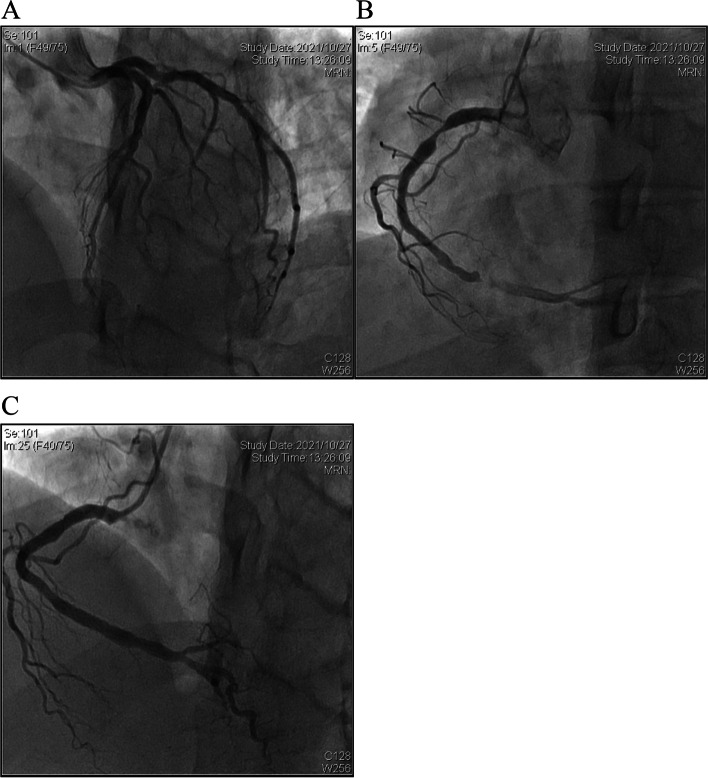
Percutaneous coronary intervention on October 27, 2021. **A** LAD patency with a stent. **B** severely stenotic RCA with 90% in the proximal segment and 99% in the distal segment. **C** after stent implantation in RCA and post-stent dilation (**C**). LAD, left anterior descending artery; RCA, right coronary artery

On October 26, 2021, the patient was hospitalized again due to typical exertional angina. Percutaneous coronary angiography showed patency LAD with a stent (Fig. 3A) and severely stenotic RCA (90% in the proximal and 99% in the distal segment) (Fig. 3B). Two GuReater stents (4.5 mm × 12 mm and 4.5 mm × 21 mm) were successfully implanted (Fig. 3C). After implanting the stent in the proximal segment, a posterior expansion balloon (Quantum Maverick, 4.5 mm × 12 mm) was used under a relatively high pressure of 16–24 atm to ensure effective apposition of the stent. Finally, intravascular ultrasound was performed which showed good apposition of the stent in the proximal segment of RCA, without hematoma or dissection. The patient had no symptoms of angina pectoris when a telephone follow-up was performed on March 23, 2022.

**Fig. 4:**
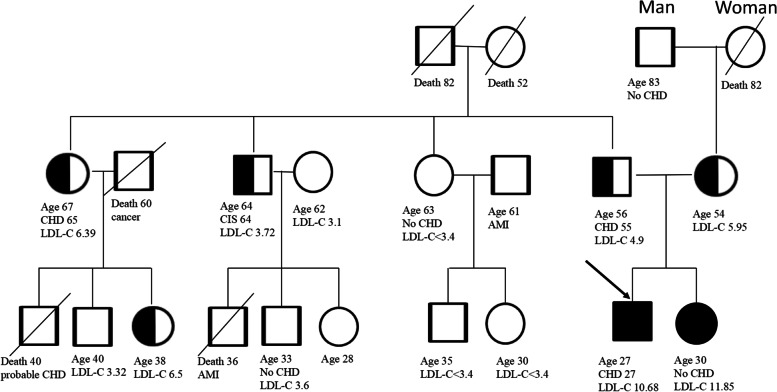
Family tree of proband (arrow) and his elder sister. LDL-C, low-density lipoprotein cholesterol (mmol/L); CHD, coronary heart disease; CIS, cerebral ischemic stroke; AMI, acute myocardial infarction

We conducted a family screening and created a family tree (Fig. 4). The patient’s father, mother, and elder sister had high LDL-C levels, especially his elder sister, whose LDL-C was as high as his. His father had received coronary artery bypass grafting at the age of 55. His aunt had received two stents planted in the RCA because of myocardial infarction (MI) at the age of 65, and his uncle had experienced cerebral infarction at the age of 64. In addition, one of his cousins had died of MI at the age of 36, and another cousin who had diabetes had died at the age of 40.

**Fig. 5:**
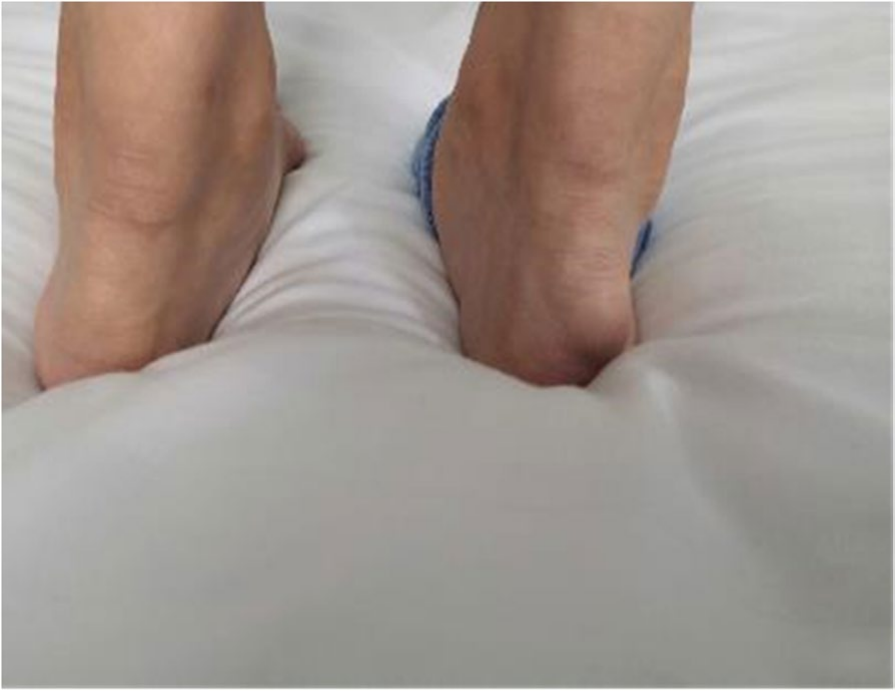
Significant Achilles tendon xanthoma of the proband’s sister

In June 2020, when the proband was admitted to the hospital, we performed relevant cardiovascular examination and evaluation to his 30-year-old elder sister. She and the proband had almost the same experience, including swelling and pain in her lower limb joints since adolescence, xanthoma of the Achilles tendon (Fig. 5) and corneal arch, the same or even higher plasma cholesterol levels (TC 14.31 mmol/L and LDL-C 11.85 mmol/L), little and limited medication, and similar light eating habits. However, she had not experienced any symptoms of angina. She was diagnosed with FH according to the DLCN score of 15 (eight points for LDL-C > 8.5 mmol/L; six points for xanthoma of Achilles tendon; and one point for hypercholesterolemia family history). CCTA showed that the CAC was just 0.5 (in the proximal segment of LAD) and no plaque or stenosis was found in any of the coronary arteries. Ultrasound showed atherosclerotic plaques (thickness 1.3–1.5 mm) in the carotids bilaterally.

To determine an LDL-C reduction strategy suitable for the family, she iniallty received subcutaneous evolocumab 420 mg, initially without any oral medicine, to observe the LDLR upregulation effect by evolocumab in the context of her family’s genetic background. Accomplishing this could provide guidance for her brother’s follow-up treatment and necessary treatment for her. Unfortunately, the LDL-C levels only reduced by 18.1% after 2 weeks. Subsequently, rosuvastatin 20 mg and ezetimibe 10 mg were taken orally every day as the main LDL-C lowering treatment for her, and evolocumab 140 mg was injected subcutaneously every 2–3 weeks. The results showed that oral medications greatly improved the LDL-C lowering effect of evolocumab. A maximum reduction of LDL-C (73.4%) was reached on October 24, 2020 (Table 2).

**Table 2:** Blood lipid levels and lipid-lowering strategies of the sister

Date	TC	LDL-C	Lp(a)	Lipid-lowering strategies	Notes
6-24-2020	14.31	11.85		evolocumab 420 mg SC	baseline levels without any medication
6-30-2020	11.66 (−18.5%)	9.76 (−17.6%)	112		6 days after evolocumab 420 mg
7-7-2020	11.5 (−19.6%)	9.71 (−18.1%)			13 days after evolocumab 420 mg
7-14-2020	11.87 (−17.0%)	11.27 (−4.9%)		rosuvastatin 20 mg qd ezetimibe 10 mg qd	20 days after evolocumab 420 mg
7-29-2020	7 (−51.1%)	4.65 (−60.8%)	136.6 (+ 22.0%)	rosuvastatin 20 mg qd ezetimibe 10 mg qd	
8-22-2020	5.62 (−60.8%)	3.67 (−69.0%)	113.3 (+ 1.2%)	rosuvastatin 20 mg qd ezetimibe 10 mg qd	
9-12-2020				evolocumab 140 mg SC rosuvastatin 20 mg qd ezetimibe 10 mg qd	
10-12-2020	6.6 (−53.9%)	4.39 (−63.0%)	95.7 (−14.6%)	rosuvastatin 20 mg qd ezetimibe 10 mg qd	
10-14-2020				evolocumab 140 mg SC rosuvastatin 20 mg qd ezetimibe 10 mg qd probucol 0.5 g bid	
10-24-2020	4.94 (−65.5%)	3.15 (−73.4%)	90.5 (−19.2%)	rosuvastatin 20 mg qd ezetimibe 10 mg qd probucol 0.5 g bid	10 days after evolocumab 140 mg
10-28-2020				evolocumab 140 mg SC rosuvastatin 20 mg qd ezetimibe 10 mg qd probucol 0.5 g bid	
11-28-2020	5.46 (−61.8%)	3.85 (−67.5%)	90.2 (−34%)	rosuvastatin 20 mg qd ezetimibe 10 mg qd probucol 0.5 g bid	
3-13-2021	6.28 (−56.1%)	4.56 (−61.5%)	89.4 (−20.2%)	rosuvastatin 20 mg qd ezetimibe 10 mg qd probucol 0.5 g bid	
4-17-2021	6.36 (−55.6%)	4.46 (−62.4%)	93 (−17.0%)	rosuvastatin 20 mg qd ezetimibe 10 mg qd probucol 0.5 g bid	

The levels and percent of change from baseline are expressed as mmol/L (%) for TC and LDL-C and mg/dL (%) for Lp(a)

*TC* Total cholesterol, *LDL-C* Low density lipoprotein cholesterol, *Lp(a)* Lipoprotein (a), *LA* Lipoprotein apheresis, *qd* Once a day, *bid* Twice a day, *SC* Subcutaneous

The patients agreed to the our treatment plan and followed the instructions after receiving thorough explanations regarding their conditions. No other adverse nor unanticipated events were identified in the proband and his elder sister at the latest follow-up on March 23, 2022.

**Fig. 6:**
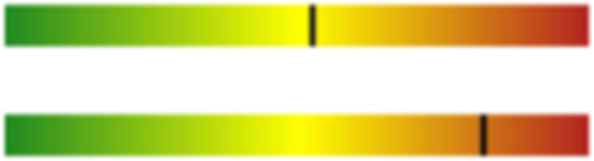
The proband’s lipoprotein subclasses analyzed by nuclear magnetic resonance. The average size of the LDL particle was 19.9 nm (upper strip), and the LDL-C/LDL particle discordance (strip below) was 13.6%. LDL, low-density lipoprotein; LDL-C, low-density lipoprotein cholesterol

**Fig. 7:**
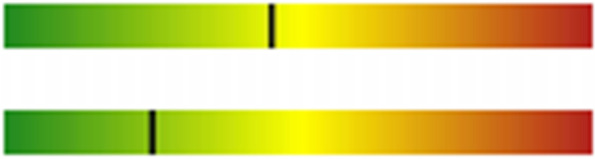
The sister’s lipoprotein subclasses analyzed by nuclear magnetic resonance. The average size of the LDL particle was 20.9 nm (upper strip) and the LDL-C/LDL particle discordance (strip below) was 0.1%. LDL, low-density lipoprotein; LDL-C, low-density lipoprotein cholesterol

We analyzed the lipoprotein subclasses using nuclear magnetic resonance before lipid lowering therapy to determine the possible causes of different clinical manifestations in the two siblings. The results showed that the LDL particle (LDL-P) size of the proband was smaller than that of his sister, and the LDL-C/P discordance of the proband was much greater than that of his sister (Figs. 6 and 7) [3].

## Discussion and conclusions

### Risk factors other than genetics and LDL-C levels

Gene mutation is undoubtedly the main factor for increased LDL-C levels in siblings. FH usually results from a loss-of-function mutation in the *LDLR* gene or mutations in the apolipoprotein B (*APOB*) gene that reduces the binding of APOB-containing lipoproteins to the LDLR or a gain-of-function mutation in the *PCSK9* gene [8, 9]. The loss-of-function mutation in the LDL receptor adaptor protein 1 (*LDLRAP1*), a rare autosomal recessive form, has also been recorded [10]. More than 1600 mutations in the *LDLR* gene have been identified and are available for review in more than 90% of patients with FH, while those in the *APOB* gene account for 2–5% of cases in northern Europe [11, 12]. Mutations leading to gain-of-function of PCSK9 activity have been identified in less than 5% of cases of FH in most areas [13, 14]. Autosomal recessive hypercholesterolemia with mutations in the *LDLRAP1* gene is much rarer, and most reported patients are from Lebanon and Sardinia [15, 16]. Although genetic testing may be valuable to determine the type of mutation, its benefit with regard to choosing the lipid-lowering treatment and reducing cardiovascular risks is limited, as nearly 15% of the patients with autosomal dominant FH test negative for mutations in any of the three identified genes described above [11]. There is no strong evidence to support the notion that specific genetic mutations’ influence on cardiovascular diseases differs from others [17, 18]. The established guidelines for FH diagnosis do not depend on genetic testing. Instead, it is primarily the phenotype (the degree of hypercholesterolemia and years of exposure to high cholesterol) that determines vascular risk. Therefore, patients are treated based on their LDL-C levels rather than their genotype. In this proband, genetic testing was recommended to the family. However, financial problems limited the ability to perform the test.

In addition to genetics, the siblings had other shared risk factors such as exposure to a similar degree and duration of hypercholesterolemia. Patients with FH have a 3–20-fold greater risk of premature ASCVD than individuals without FH [3, 19–22]. However, the prevalence rate of ASCVD in patients with FH with the same genetic background varies greatly [23–25]. Despite LDL-C elevation being unequivocally the main determinant of CVD risk in FH, significant variability in the incidence of CVD events has been reported in patients with FH, even among those carrying the same genetic mutations and comparable LDL-C levels [26]. In the context of high cholesterol levels, other traditional cardiovascular risk factors such as old age, male sex, family history of premature ASCVD, high blood pressure, increased body mass index, active smoking, and lipoprotein (a) [Lp(a)] levels are more harmful and independent predictors of an increased risk of incident ASCVD in patients with FH [27, 28]. In general, the types of risk factors in patients with FH appear similar to those of traditional risk factors (e.g., Framingham) [29, 30], but contributions to ASCVD are different in every aspect. Framingham Risk Score is not recommended in patients with FH as it underestimates the risk due to long-term exposure to high cholesterol levels. The SAFEHEART is a multicenter, nationwide, long-term prospective cohort study of a molecularly defined population with FH with or without previous ASCVD in Spanish; the SAFEHEART study presented a new equation that is able to prospectively assess incident ASCVD risk over 5 and 10 years in patients with FH [28]. In this proband, male sex, three to five cigarettes per day, and smaller LDL-P size are possible reasons for the different outcomes. Elevated Lp(a) levels are present in 30–50% of patients with HeFH. Elevated Lp(a) will further increase the risk of premature ASCVD in patients with FH. The elevation of FH and Lp(a) is independently associated with premature ASCVD, because the genetic basis of FH and high Lp(a) is different [31]. Obviously, the level of Lp(a) is not a cause for different severity of coronary lesion between the siblings as the level of Lp(a) of the elder sister is higher than that of her younger brother.

### LDL particle

Male sex and smoking were considered the main reasons for different clinical phenotypes between two siblings, but underlying mechanism remained unclear. The LDL-P size may be a potential reason. In general, LDL-C level can be used to represent LDL level. However, when the LDL-C level is inconsistent with the LDL-P concentration, the clinical and subclinical outcomes track with LDL-P more than with LDL-C [32, 33]. The smaller the LDL-P size is, the higher the LDL-P concentration is at the same LDL-C level. In this pair of FH cases, the LDL-C/P discordance of the proband is much greater than that of his elder sister (the LDL-C/P discordance of the proband was 13.6%, and that of the elder sister was 0.1%). In the proband, the average size of the LDL-P is 19.9 nm, while that of the elder sister is 20.9 nm; this supports the hypothesis that small LDL-P in patients with FH may lead to earlier and more severe cardiovascular events. Therefore, the LDL-P size may be the key factor leading to the difference. LDL-P size has the potential to be a new indicator for predicting the prognosis of FH and non-FH populations, although this still requires large-scale prospective studies to verify their association. Furthermore, subsequent studies should focus on the potential of developing drugs that increase the particle size of LDL-P to reduce cardiovascular events.

### PCSK-9 inhibitor

A triple treatment regimen of statins, ezetimibe, and PCSK9 inhibitor was recommended to the siblings. PCSK9 inhibitors are a class of novel lipid-lowering drugs whose main mechanism is preventing PCSK9 from binding to the LDLR. The inhibition of this binding reduces the degradation of LDLR, increases the uptake of LDL by LDLR from the bloodstream, and ultimately reduces plasma LDL-C concentration [34]. PCSK9 inhibitors have been approved by the Food and Drug Administration and used primarily in the treatment of FH and patients who were not able to achieve LDL-C therapeutic goals with common lipid-lowering drugs or who are statin intolerant.

In this pair of FH cases, a PCSK9 inhibitor was used in combination with statins and ezetimibe to intensify therapy and achieve therapeutic goals. Statins lead to up-regulation of LDLR by inhibiting intracellular cholesterol synthesis, whereas PCSK9 inhibitor can increase the number of LDLR by inhibiting the degradation of LDLR [35]. Therefore, the ability of PCSK9 inhibitor to reduce LDL-C is also closely related to the function of LDLR. In this study, when the proband’s elder sister used evolocumab alone, the LDL-C lowering effect was not significant. However, when combined with statin and ezetimibe, the LDL-C lowering effect was considerably improved. The possible explanation is evolocumab increase the number of LDLR, yet the function of LDLRs were defective. Statins upregulate LDLR, therefore their combination with evolocumab could further increase the number of LDLRs. This further increase in number could compensate for functional defects to a certain extent. This suggests that PCSK9 inhibitors combined with statins are particularly important for patients with HeFH.

The effect of evolocumab on the LDL-P size has not been determined in the present study. It is reported that PCSK9 monoclonal antibody could reduce both smaller and larger LDL-P and appeared to be more effective in reducing larger LDL-P [36, 37]. The cholesterol content of LDL is positively correlated with the particle size. Therefore, PCSK9 inhibitors can reduce LDL-C more efficiently due to their characteristic of clearing large LDL-P.

Patients with FH have a much higher risk of premature ASCVD, but the clinical manifestations are heterogeneous. In current clinical practice in China, the combination of PCSK9 inhibitor, statins, ezetimibe, and lipoprotein apheresis is an effective strategy to effectively reduce the LDL-C levels in patients with FH of severe clinical phenotype. Male sex was the main risk factor for different clinical outcomes in this pair of FH cases, and the smaller LDL-P size might be the potential mechanism. Therefore, this suggests further studies are warranted regarding the relationship between particle size and cardiovascular events. Additionally, finding new predictors of cardiovascular events in patients with or without FH would be helpful.

## Data Availability

All datasets generated for this study are included in the manuscript.

## Abbreviations

APOB: Apolipoprotein B
ASCVD: Atherosclerotic cardiovascular disease
CAC: Coronary calcium score
CCTA: Coronary computed tomographic angiography
CHD: Coronary heart disease
DLCN: Dutch Lipid Clinic Network
FH: Familial hypercholesterolemia
HeFH: Heterozygous familial hypercholesterolemia
LAD: Left anterior descending
LDL-C: Low density lipoprotein cholesterol
LDLR: Low density lipoprotein cholesterol receptor
LDLRAP1: Low density lipoprotein cholesterol receptor adaptor protein 1
LDL-P: Low density lipoprotein cholesterol particle
Lp(a): Lipoprotein (a)
MI: Myocardial infarction
PCSK9: Proprotein convertase subtilisin/kexin type 9
RCA: Right coronary artery
TC: Total cholesterol
